# The Refusal of Food in Infancy

**Published:** 1908-06-20

**Authors:** 


### Diseases of Children.

### THE REFUSAL OF FOOD IN INFANCY.

Not infrequently infants are brought for consultation because they refuse to take the breast or the bottle, and will even struggle against spoon-feeding. If by persistence the food is got down, it is liable to be at once vomited.

At birth breast-feeding may be a mechanical difficulty on account of small and retracted nipples. In exceptional cases the nipples are supposed to be too large. Occasionally the child presents gross structural defects, such as cleft palate, inflammations of the gums, mouth, or fauces, or respiratory obstruction in consequence of snuffles, adenoids, or enlarged tonsils. These affections are equally important in bottle-feeding. Here, too, the fault may be in the teat, which may be too large, but is more often too small, of a wrong shape, and with an orifice so minute that the infant has to over-exert itself to obtain the food. A proper teat is roughly the shape of the thumb of a glove, of rubber, and capable of easy inversion for cleaning.

In rare cases there is no obvious explanation for the refusal of the breast or bottle, and one is driven to a vague diagnosis of imperfect co-ordination or imperfect development of the nervous centres for suction and deglutition. Pinard reported a remarkable case in which the reflex area for suction receded gradually from the tip to the base of the tongue. The child died from hydrocephalus. In a few instances of no apparent causation the infant will suddenly begin to suckle normally. Frequently the fault lies in the flavour or the composition of the food. Occasionally the refusal depends on mental defect or upon gross disease. Thus, I have seen it in an infant which eventually proved to have general tuberculosis, although there was no post-mortem evidence of any glandular or other pressure which would have interfered with swallowing. It was apparently due to complete anorexia. Such anorexia may occur in many diseases. Palatal palsy is another case, but this is rare in infancy.

Another source of trouble is one which can easily be avoided if one can get the mother or nurse to carry out the simple but necessary treatment. A case in point is that of a boy, aged eighteen months, who was fed on a mixture of milk, cream, water, and lactose, and would take no solid food. He was very fat, of a good colour, intelligent, and had sixteen teeth. His liver was unduly large, and he could not stand. There was no doubt that the refusal to take solid food was due to the prolonged continuance of bottle-feeding. I saw him again thirteen months later for the same reason, namely, that he would not take solid food. The explanation was equally obvious, namely, that the advice previously given had not been followed, for he was still entirely bottle-fed.

It may be stated that a child can be brought up for the first two or three years of life on a milk diet without suffering the least in health; but it is doubtful whether it is ever advisable to permit such a mode of feeding. It is much better to accustom the child gradually to different foods, for abrupt changes are liable to derange the alimentary tract, which requires training in the development of its functions, just as does any other system of the body.

The treatment of patients of this type consists in stopping the bottle absolutely and permanently. If bottle-feeding is partially discontinued towards the end of the first year of life, the difficulty does not arise. If it has arisen, the bottle must be prohibited entirely until starvation compels the child to take from a cup or spoon. In the case quoted the mother was neurotic, and the nurse a silly creature who had not the strength of will to carry out the treatment, as she could not bear to hear the child cry, and was afraid it might come to some harm from want of food.

A similar instance in a girl of eighteen months was quickly cured by this simple commonsense method in the course of a few days, for the nurse was a good one. Curiously, in both children there was a strong neurotic history, and I am convinced that this symptom may be taken as one of the stigmata of a neurotic disposition in infancy. It may take a more alarming form in that food is well taken, provided that it is absolutely smooth, and not the least thick, whereas vomiting or refusal of food will follow the thickening of the diet by nursery rusk, or, indeed, the presence of a few crumbs.

Occasionally it happens that a baby for no apparent reason will, after taking more or less solid food for some considerable time, suddenly refuse everything but milk, and will vomit everything else which it is forced to take. Such a case was that of a boy, aged two years, who had had scurvy in the first year of life, and later on severe broncho-pneumonia. Recovery was complete, and he was gaining weight rapidly when the above symptoms came on. He rapidly lost weight, nine ounces in one week and thirteen in the next five days. There was no apparent cause. He seemed quite bright and cheerful, and slept well. On a diet of milk and milk sugar he is improving, and will no doubt return to his ordinary food habits. Such cases are distinctly alarming to the mother, and a source of anxiety to the doctor.

A rapid loss of weight may indicate the incubation period of one of the specific fevers, or it may indicate tuberculosis. The vomiting is suggestive of tuberculous meningitis. Careful inquiry will generally show that the vomiting is deliberate on the part of the child. He makes up his mind to be sick, even before the food has been taken. This, too, may be regarded as a neurotic symptom and called "habit-vomiting," provided care is taken to exclude other possible causes, especially gastric disorders. Change of surroundings is more important than drugs, and temporary starvation has never in my experience proved harmful. The vomiting ceases, and the loss of weight is rapidly regained as the child's appetite returns.

